# Fever as the Sole Presentation of Giant Cell Arteritis: A Near Miss

**DOI:** 10.1177/2324709619850222

**Published:** 2019-06-11

**Authors:** Pooja Poudel, Thein Swe, Michael Wiilliams, Eduardo Bonilla

**Affiliations:** 1 State University of New York Upstate Medical University, Syracuse, NY, USA

**Keywords:** fever of unknown origin, giant cell arteritis

## Abstract

Giant cell arteritis (GCA) presenting solely as fever is very rare. Usually, it manifests with typical features such as visual problems, headache, jaw claudication, or it can be associated with polymyalgia rheumatica. We present a case of a patient with GCA who presented only with prolonged fever. The cause of fever could not be initially identified in spite of a comprehensive workup. The patient was started on steroids for presumed GCA resulting in the resolution of fever. It is of paramount importance to consider GCA in the differential diagnosis of fever of unknown origin. Early diagnosis with effective treatment is crucial since the mortality rate remains high for untreated cases.

## Introduction

Giant cell arteritis (GCA) represents the most common primary vasculitis in persons older than 50 years.^1^ It usually involves large and medium-sized arteries. It typically presents with a headache, jaw claudication, visual changes, and symptoms of polymyalgia rheumatic.^2^ Rarely, it may present with fever as the only dominant symptom. When it is not associated with other typical GCA features, it can lead to delay in diagnosis. The definitive diagnosis of GCA is through a temporal artery biopsy, which reveals multinucleate giant cells and fragmented internal elastic membrane with lymphocyte and macrophage infiltration most pronounced in the tunica media. We report a case of an 86-year-old male who presented with fever of unknown origin (FUO) in absence of other typical features.

## Case Presentation

An 86-year-old male with no significant past medical history was admitted to the hospital with a history of prolonged high fever unto 104°F. Workup for infection was unremarkable. He did not have leukocytosis, white blood cell count was 5.2/mm^3^, and chest X-ray, computed tomography (CT) scan of abdomen, and transthoracic echocardiogram were unremarkable. Blood culture and urine culture showed no growth, and flu screen was negative. Since the patient had elevated D-dimer, Doppler ultrasound was ordered with the suspicion of thrombus, which could explain the fever but it showed no evidence of deep vein thrombosis. His ferritin level was elevated at 725 ng/mL (reference range: 26-388 ng/mL), hemoglobin 12.0 g/dL, erythrocyte sedimentation rate (ESR) was 66 mm/h (ref: <20 mm/h), and C-reactive protein (CRP) 55.9 mg/L (ref: <8 mg/L). He was found to have a positive ANA speckled pattern 250, but additional laboratory findings including ANA specificity, rheumatoid factor, and ANCA were found to be negative. During the hospital course, he started having episodes of confusion and there was suspicion of meningoencephalitis. Physical examination was not indicative for meningitis with negative meningeal signs, and lumbar puncture was done, which was unremarkable for viral and bacterial pathogens. Rapid plasma reagin, hepatitis panel, QuantiFERON, mono spot, HIV test, and paraneoplastic panel tests were negative.

**Figure 1.**
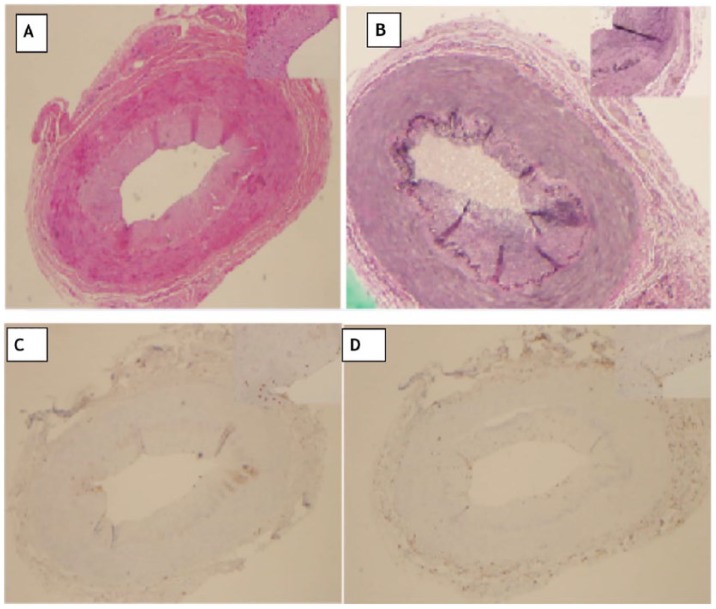
(A) Hematoxylin and eosin staining at 20× (inset at 40× showing mononuclear infiltrate within the intimal layer). (B) EVG staining at 20× (inset at 40× showing disruption of elastin layers). (C) CD3 staining at 20× (inset at 40× showing lymphocytes within the intimal layer). (D) CD163 staining at 20× (inset at 40× showing histocytes within the intimal layer). Pathology section: Sections in the figure show a vessel with moderate intimal hyperplasia with inflammatory cells present within the intimal layer. An elastin stain was performed showing a disruption of the elastic layer of the intimal surface with fibrosis. CD3 and CD163 were performed and revealed the presence of lymphoid and histiocytes. This may represent the previous arteritis that occurred.

Malignancy was ruled out with normal CT scan of chest, abdomen, and pelvis and magnetic resonance imaging of brain. Bone marrow biopsy showed changes suspicious for myelodysplastic syndrome with single lineage dysplasia (refractory anemia), but he did not have neutropenia or leucopenia. There was low suspicion of GCA initially as he denied typical symptoms of GCA such as headache, jaw pain/claudication, stiffness in the shoulder joints, or visual disturbances. With no explanation for the findings of fever, he was started on prednisone 60 mg for presumed GCA after which his condition markedly improved and fever was resolved. He had temporal artery biopsy of both sides, of which biopsy of the right side showed mild to moderate intimal hyperplasia, partial disruption of internal elastic lamina with fibrosis, and minimal lymphocytic and histolytic infiltrates, which was consistent with GCA (Figure 1).

## Discussion

Workup of FUO can be difficult at times when patients present with atypical clinical symptoms and have nonspecific laboratory findings. Certain FUOs have characteristic fever patterns that can be helpful in the absence of specific laboratory tests and that includes Still’s disease, visceral leishmaniasis (double quotidian fever), infectious disease, or lymphoma (hectic septic fever).^3,4^ FUO was first defined by Petersdorf and Beeson in the year 1961,^5^ and at that time, it was mostly attributable to an infectious disease followed by malignancies and then to connective tissue diseases (CTDs).^5^ Nowadays, CTDs are less frequently diagnosed as FUO because of the availability of serologic test available to diagnose most of these disorders. So clinicians are able to establish a diagnosis before the criteria of FUO is met. Examples of CTD that do not have specific test include late-onset rheumatoid arthritis, polymyalgia rheumatic, or GCA, all of which remain an important cause of FUO.^3,5^

GCA is a chronic granulomatous vasculitis, which is mostly seen in the extracranial branches of carotid arteries. As the disease is systemic, widespread vascular involvement is commonly seen. It is rarely seen in individuals younger than 50 years of age,^1^ usually peaks in the seventh decade,^5^ and has a slight male predominance. It is mostly seen in the white population. The diagnosis should be suspected in the patients who are older than 50 years and who are found to have one of the signs or symptoms shown in Table 1.

**Table 1.** Clinical features of GCA.

1	New headache
2	Sudden onset of visual disturbances, especially transient monocular visual loss
3	Jaw claudication
4	Unexplained fever, anemia, or constitutional signs or symptoms
5	High ESR and/or CRP

Abbreviations: ESR, erythrocyte sedimentation rate; CRP, C-reactive protein.

In a study of 535 patients undergoing temporal artery biopsy for suspected GCA, the combination of 4 findings correlated with a 95% probability of a negative temporal artery biopsy^3^ (Table 2).

**Table 2.** 

1	A normal or mildly elevated ESR (less than 40 mm/h)
2	Absence of jaw claudication
3	Absence of temporal artery tenderness
4	The presence of synovitis, suggesting an alternative diagnosis

Abbreviation: ESR, erythrocyte sedimentation rate.

Our patient did not have the findings in Table 2, except for elevated ESR, which can also be elevated in infections, so there was low suspicion of GCA initially.

Although fever can present along with other typical features such as jaw claudication, jaw pain, headache, polymyalgia symptoms, FUO as the only presentation of GCA is described infrequently.^6,7^ The gold standard of diagnosis is temporal artery biopsy that can be falsely negative due to the presence of skip lesions. A larger section (4-6 cm) of the vessel has to be excised and multiple sections need to be studied. Characteristic histopathologic findings of GCA includes panarteritis, fragmentation of internal elastic lamina along with infiltration of CD4+ lymphocytes, macrophages, and giant cells. Giant cells are common but not required for diagnosis. Color Doppler ultrasonography of the temporal arteries can be done before biopsy, which is more specific for the diagnosis.^8^ Although traditionally ESR and CRP can be elevated in GCA, it is also increased on malignancy, infection, or other CTD. Conversely, lower values do not exclude the possibility of GCA. If there is a high suspision of GCA and there is no pain in the temporal artery, FDG-PET/CT is performed which shows increased uptake in the wall of the arteries.^9^ In a study of 167 patients with biopsy-proven GCA, the ESR was less than 50 in 11% and less than 40 in 5%.^10^ Multisystemic disease is one of the most frequent causes of FUO in elderly patients. The investigation of the elderly patients with FUO should include temporal artery biopsy if classic tests such as blood count, urinalysis, chemistries, cultures, and imaging do not yield any clue.^11^ Treatment with high-dose steroids should be promptly started once we suspect the diagnosis of GCA.^2^ We should obtain temporal artery biopsy as soon as possible, but the treatment should not be withheld while awaiting the procedure as the use of the steroids for at least 2 weeks will not affect the biopsy result.^12^ In patients who are at high risk of adverse effects of steroids, steroids sparing agents such as tocilizumab^13^ or methotrexate^14^ should be considered. In the patients with visual complications, intravenous methylprednisolone can be given as a pulse therapy.^12^

Calamia and Hunder published a study of clinical characteristics of 100 patients with GCA who were biopsy-proven and found that symptoms were diverse and often did not lead to definitive diagnosis^15,16^ (Table 3).

**Table 3.** Temporal Arteritis: Symptoms in 100 Patients.^15,16^

Symptoms	Total Number of Patients
Weight loss or loss of appetite	50
Malaise or fatigue	40
Fever	42
Polymyalgia rheumatica	39
Other musculoskeletal pains	30
Joint pain and swelling	15
Symptoms related to arteries	83
Headache	68
Visual symptoms	
Transient	16
Fixed	14
Jaw claudication	45
Swallowing claudication or dysphagia	8
Tongue claudication	6
Limb claudication	4
Signs related to arteries	66
Artery tenderness	27
Decreased temporal artery pulsations	46
Erythematous, nodular, or swollen scalp arteries	23
Large artery bruits	21
Decreased large artery pulses	7
Visual loss	14
Ophthalmoscopic abnormalities	18
Raynaud’s phenomenon	3
Central nervous system abnormalities	15

## Conclusion

Giant cell arteritis may rarely present with FUO as the only manifestation. It is of paramount importance to consider the diagnosis of GCA in the differential diagnosis of a patient presenting only with FUO. This is particularly important since early treatment with steroids can prevent potential irreversible complications from the disease.
